# Comparison of qPCR and metabarcoding for environmental DNA surveillance of a freshwater parasite

**DOI:** 10.1002/ece3.11382

**Published:** 2024-05-06

**Authors:** Mark Johnson, Sasha Tetzlaff, Aron Katz, Jinelle Sperry

**Affiliations:** ^1^ Engineer Research and Development Center Champaign Illinois USA; ^2^ Illinois Natural History Survey, Prairie Research Institute University of Illinois Urbana‐Champaign Champaign Illinois USA; ^3^ Department of Entomology University of Illinois Urbana–Champaign Urbana Illinois USA; ^4^ Department of Natural Resources and Environmental Sciences University of Illinois Urbana–Champaign Urbana Illinois USA

**Keywords:** eDNA, metabarcoding, occupancy modeling, qPCR, *Salmincola edwardsii*

## Abstract

Analysis of environmental DNA (eDNA) has been successfully used across freshwater ecological parasitology to inform management of ecologically and economically important species. However, most studies have used species‐specific quantitative polymerase chain reaction (qPCR) assays to detect target taxa. While generally effective, this approach limits the amount of community and management‐supporting data that can be obtained from eDNA samples. If eDNA metabarcoding could be conducted with the same accuracy of a single species approach, researchers could simultaneously detect a target species while obtaining vast community data from eDNA samples. We sampled 38 freshwater sites on Fort McCoy, Wisconsin and compared qPCR to metabarcoding for eDNA detection of the ectoparasitic gill louse *Salmincola edwardsii*, an obligate parasite of *Salvelinus* fishes (chars). We found no evidence to suggest *S. edwardsii* occupancy or detection probabilities differed between qPCR and metabarcoding. Further, we found that the number of *S. edwardsii* reads from metabarcoding were negatively predictive of *C*_*T*_ values from qPCR (*C*_*T*_ value indicates cycle a significant amount of target eDNA is detected, with lower *C*_*T*_s indicative of more DNA), demonstrating that our metabarcoding reads positively predicted qPCR DNA quantities. However, the number of reads was not predictive of overall qPCR score (number of positive qPCR replicates). In addition to *S. edwardsii* , metabarcoding led to the detection of a vast community of over 2600 invertebrate taxa. We underscore the necessity for conducting similar analyses across environments and target species, as the ecology of eDNA will vary on a per‐study basis. Our results suggest that eDNA metabarcoding provides a highly sensitive and accurate method for detecting parasitic gill lice while also illuminating the broader biological community and co‐occurrence of species in the environment.

## Graphical abstract

We sampled 38 freshwater sites on Fort McCoy, Wisconsin and compared qPCR to metabarcoding for eDNA detection of the ectoparasitic gill louse *Salmincola edwardsii*, an obligate parasite of *Salvelinus* fishes (chars). We found no evidence to suggest *S. edwardsii* occupancy or detection probabilities differed between qPCR and metabarcoding. Our results suggest that eDNA metabarcoding provides a highly sensitive and accurate method for detecting parasitic gill lice while also illuminating the broader biological community and co‐occurrence of species in the environment.

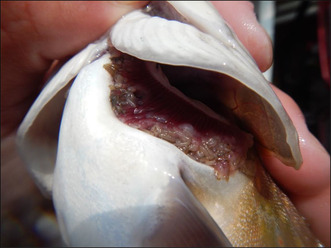

## 1 INTRODUCTION

Freshwater fish populations around the world have become increasingly threatened as a result of increased stressors in their natural environment, such as invasive species, habitat loss, and climate change (Magurran, 2009). Parasites are one such stressor that influences native freshwater fish communities, impacting survival, reproduction, and behavior (Barber et al., 2000; Sindermann, 1987). While a natural part of an ecosystem network, recent evidence suggests that emerging threats, such as climate change and increased invasions, will exacerbate the negative effects of parasites on freshwater fish (Custodio da Costa et al., 2021; Marcogliese, 2008). With this predicted increase in parasitic impacts and threats to freshwater fish, it is vital to have methods that accurately and rapidly detect fish parasites in their natural habitat. Conventional approaches that largely depend on surveying captured fish are often time consuming, costly, detect parasites in low numbers or at certain life stages, and reactive instead of proactive (Bass et al., 2015; Sieber et al., 2020). Furthermore, internal parasite detections that require fish capture and assessment are often lethal, further stressing vulnerable host populations (Duval et al., 2021). To address these limitations, researchers and natural resource managers are increasingly turning to environmental DNA (eDNA) to detect freshwater parasites (Bass et al., 2015; Duval et al., 2021; Katz et al., 2023).

In this context, eDNA refers to genetic material that is shed from an organism into its environment (Barnes & Turner, 2016; Thomsen & Willerslev, 2015). The eDNA is collected and analyzed from bulk environmental samples such as aquatic (Closek et al., 2019; Deiner & Altermatt, 2014; Gold et al., 2021; Pont et al., 2018), sediment (Alsos et al., 2018; Katz et al., 2021; Yoccoz et al., 2012), and air (Clare et al., 2022; Johnson, Cox, et al. 2021; Lynggaard et al., 2022) to determine whether that sample contains genetic material from one or multiple species of interest. Bass et al. (2015, 2023) reviewed the applications of eDNA specifically for detecting parasites, highlighting its potential for effectively detecting parasites in natural systems and demonstrating that eDNA often outperformed conventional surveying methods. While eDNA has shown success in detecting freshwater parasites, the majority of studies have focused on single species identification using quantitative polymerase chain reaction (qPCR) based methods (Huver et al., 2015; Katz et al., 2023; Richey et al., 2018; Rusch et al., 2018; Sengupta et al., 2019; Sieber et al., 2020). This species‐specific approach has been valuable, but advancing technology has paved the way for whole community analysis through eDNA metabarcoding. For instance, Thomas et al. (2021) used metabarcoding to detect nematode and platyhelminth parasites in New Zealand, highlighting the potential of this method for assessing whole communities, particularly as more reference sequence library data become available. If metabarcoding could serve to detect target parasite species as efficiently as single species qPCR analyses, such results would provide the added benefit of detecting numerous taxa that may be related to the parasite (host, competitors, etc.), additional parasites, and information on community dynamics.

The advantages and disadvantages of single‐species qPCR compared to community metabarcoding have been well documented in the literature (Dritsoulas et al., 2020; Harper et al., 2018; Ruppert et al., 2019); however, the comparison of the relative efficiencies of the two methods for parasites is yet understudied. The sensitivity of qPCR to small quantities of DNA would seemingly make it well suited for detection of parasites, particularly with the potential for sequence masking, inadequate sequencing coverage and/or PCR bias present in metabarcoding (Harper et al., 2018). qPCR may also present cost and time savings compared to metabarcoding, although the effort associated with novel qPCR assay development and validation should be considered (Thalinger et al., 2021). In contrast, metabarcoding provides broader community‐level data, sequence data to confirm the presence of a species, and is becoming increasingly more affordable with advancing technologies (Andres et al., 2022; Smart et al., 2016).

Several previous studies have explored differences in focal species detection between qPCR and metabarcoding with varied results. Dritsoulas et al. (2020) found that metabarcoding was superior to qPCR for detecting entomopathogenic nematodes in soil, while other studies have found that either qPCR or metabarcoding performed the best depending on the circumstances and goals of the project (Harper et al., 2018; Schneider et al., 2016), and still more studies have determined qPCR to be the ideal method for single species detection (Blackman et al., 2020; Lacoursiere‐Roussel et al., 2016; Simmons et al., 2015; Wood et al., 2019). Some studies have examined this relationship further, exploring how the number of sequences (reads) from metabarcoding compared to qPCR *C*_*T*_ values (cycle threshold, defined as the number of cycles required for detection) and qPCR score (number of positive qPCR replicates; Harper et al., 2018). For instance, Harper et al. (2018) found a positive relationship between great crested newt (*Triturus cristatus*) metabarcoding reads and qPCR score while Biggs et al. (2015) found no relationship between reads and qPCR score for the great crested newt. The vast spectrum of these results underscore the highly context dependent nature of this comparison, with results often depending on the system and research question of interest (Blackman et al., 2020; Harper et al., 2018).

Currently, no studies have compared qPCR to metabarcoding for the detection of freshwater parasites, of which widespread eDNA surveys have been lacking (Bass et al., 2015). Exploring the comparison of qPCR‐based methods and metabarcoding through the lens of a freshwater parasite would provide an interesting dichotomy. On one hand, qPCR‐based approaches have been successful in detecting parasitic organisms, often outperforming traditional methods (Bass et al., 2015; Rusch et al., 2018; Sengupta et al., 2019). Parasites are often rare and small within the overall environment, indicating their eDNA may also be rare, which is ideal for qPCR‐based approaches. On the other hand, metabarcoding would allow for whole community analyses, including the detection of additional parasite host species (Bass et al., 2015). Rusch et al. (2018) used qPCR to detect a monogean parasite (*Gyrodactylus salaris*) and two host fish, Atlantic salmon (*Salmo salar*) and rainbow trout (*Oncorhynchus mykiss*). This required three different qPCR assays and multiple rounds of analysis, while metabarcoding, depending on its sensitivity and taxonomic targets, could potentially determine the presence of all these species with fewer analyses and more community data. If metabarcoding is as sensitive as qPCR‐based methods for detecting aquatic parasites and could give insights into the abundance of parasitic species, the amount of information gained for conservation and management would be considerably higher.

**FIGURE 1:**
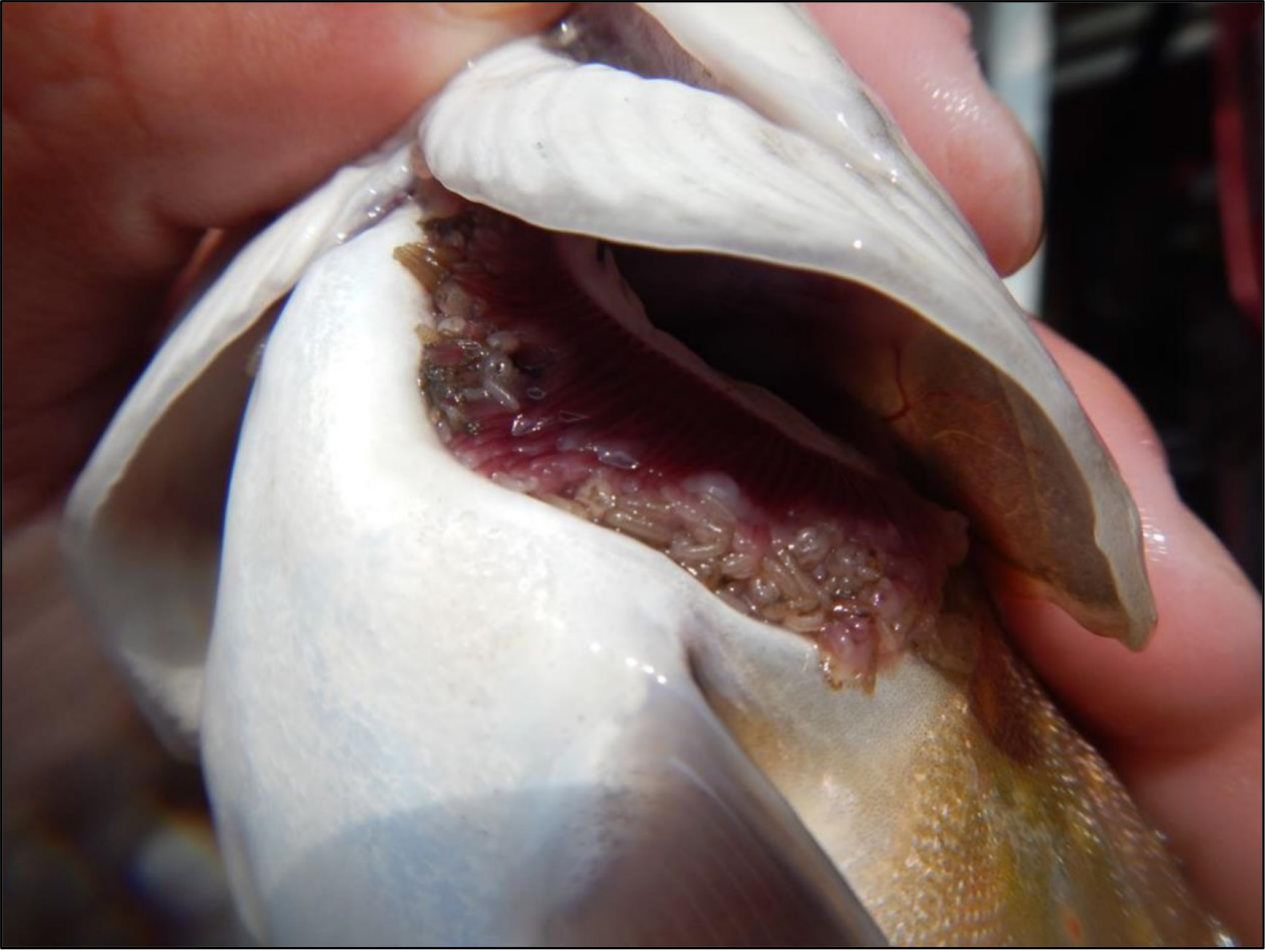
A *Salmincola edwardsii* infestation on a brook trout (*Salvelinus fontinalis*) at Fort McCoy, Wisconsin, USA. Photo Credit: John Noble.

To explore the comparison of qPCR and metabarcoding for parasite detection, we chose the ectoparasitic gill louse *Salmincola edwardsii* (Olsson 1869; Figure 1). In North America, *S. edwardsii* is a host‐specific parasite infesting the fish genus *Salvelinus*, including the economically significant native brook trout (*Salvelinus fontinalis*; Mitchill 1814; Duston & Cusack, 2002). These parasites cause physical trauma to the gills, impact respiration and swimming, and lead to mortalities of individuals (Duston & Cusack, 2002; Hasegawa et al., 2022; Mitro, 2016). Furthermore, the presence of these parasites has been shown to further exacerbate brook trout decline when in the presence of invasive brown trout (*Salmo trutta*; Mitro, 2016). Conventional methods of gill lice detection are invasive, requiring catching and collection of the host fish. Their economic importance and biology (cryptic, small, rare in the environment, and presumably low DNA shedding rate) makes *S. edwardsii* an ideal organism to compare eDNA detection methodologies and sensitivities. Recently, Katz et al. (2023) designed a novel *S. edwardsii* eDNA assay and found that qPCR was equivalent to electrofishing for detecting *S. edwardsii*. Analyzing these same samples from Katz et al. (2023) allow us to compare the sensitivities of qPCR and metabarcoding.

Here, we performed invertebrate eDNA metabarcoding on samples originally collected and analyzed for the development and validation of the *S. edwardsii* eDNA qPCR assay (Katz et al., 2023). By comparing metabarcoding detections with qPCR results, we directly assess the efficacy of eDNA metabarcoding for gill louse detection and determine how its performance compares with qPCR. This comparison explores (1) the percentage of sites that successfully detected gill lice eDNA with qPCR and metabarcoding and the occupancy and detection probabilities between methods, (2) the relationship between metabarcoding read counts, *C*_*T*_ value, and qPCR score, and (3) the pros and cons of qPCR versus metabarcoding within the context of aquatic parasite surveillance. The results of this study not only shed light on the relative performance of two widely applied eDNA survey methods but also increase our understanding of optimal eDNA approaches for the detection of economically and ecologically damaging parasites in aquatic systems.

## 2 MATERIALS AND METHODS

### 2.1 eDNA collection and extraction

**FIGURE 2:**
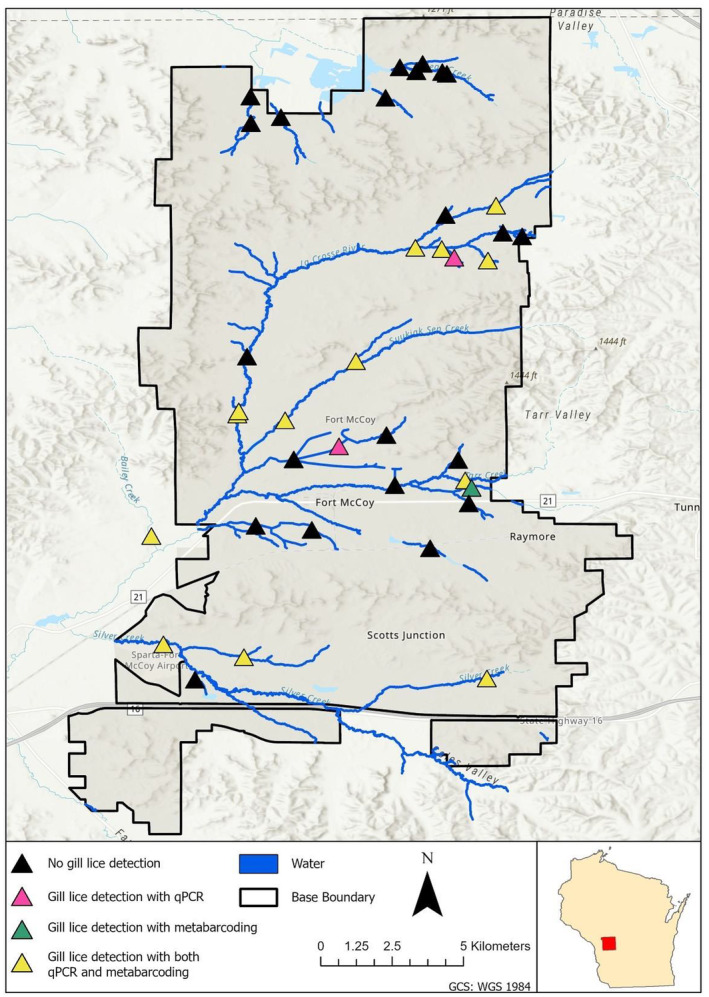
Sites on Fort McCoy, Wisconsin, USA where stream water was collected to test for the presence of *Salmincola edwardsii* environmental DNA. Colored triangles indicate whether gill lice eDNA was detected, detected only with qPCR, detected only with metabarcoding, or detected with both methods.

To assess the performance of metabarcoding compared to qPCR for gill lice eDNA detection, we used the same samples originally collected and analyzed by Katz et al. (2023). Water samples were collected from July 20–29, 2021 from 38 sites located within streams on Fort McCoy, Wisconsin, a United States Army installation (Figure 2). At each site, three replicate water samples were collected in 1‐L bottles (3 L total) from the right, left, and center channels of each stream. Sampling effort also included a 1‐L bottle of distilled water at each site to serve as a negative field control. All samples were stored on ice in coolers prior to filtration. Within 8 h of sampling, water samples were filtered using a vacuum pump through 0.80 μm cellulose nitrate filters. Filters were then stored in vials of cetyl trimethylammonium bromide (CTAB) buffer at room temperature for 30 days prior to DNA extraction. Environmental DNA was isolated from all samples (*n* = 159), including field (*n* = 38) and extraction (*n =* 7) controls, using a modified phenol‐chloroform‐isoamyl alcohol DNA extraction protocol to minimize PCR inhibitors and maximize DNA yield (Schrader et al., 2012). Extracted DNA was then stored in a −20°C freezer for 3 months until downstream qPCR and metabarcoding analyses were completed.

### 2.2 qPCR analysis

The methods and results for the qPCR portion of our comparison are described in Katz et al. (2023). In short, we utilized triplicate 20‐μL reaction volumes consisting of 3 μL of template; 10 μL of TaqMan Environmental Master Mix 2.0 (Applied Biosystems); 4 μL of sterile, molecular‐grade water; and 1 μL of each primer and probe with optimized concentrations (primers: 10 μM; probe: 8 μM). Thermocycling conditions were 95°C for 10 min, followed by 50 cycles of 95°C for 15 s and 60°C for 60 s. To quantify the amount of *S. edwardsii* eDNA present in each sample (copies/μL of extract), 10‐fold serial dilutions of synthetic *S. edwardsii* DNA (gBlocks) were used to generate 7‐point standard curves (3,000,000–3 copies/reaction) for each qPCR plate.

### 2.3 Metabarcoding library preparation and sequencing

A two‐step PCR protocol was used to construct metabarcoding libraries following the Illumina 16S metagenomic sequencing library preparation guidelines, modified for the cytochrome oxidase subunit 1 mitochondrial gene (COI), using the ANML invertebrate‐specific primers (LCO1490/COI‐CFMRa) developed by Jusino et al. (2019), which target a 181–187 bp region of COX1, and IDT for Illumina UD Indexes (Table 1). Locus‐specific primers, modified with Illumina overhang adapter sequences, were used for the first PCR (PCR1). Each 25 μL PCR1 reaction was performed in triplicate for all samples (*n* = 159), negative PCR controls (sterile, molecular grade water), and positive PCR controls consisting of genomic DNA isolated from gill lice tissue samples (0.1 ng/μL). Each PCR1 reaction used 25 μL volumes consisting of 5 μL of DNA template, 12.5 μL of KAPA HiFi HotStart ReadyMix (Roche Sequencing), 0.5 μL of bovine serum albumin (BSA), 4 μL of sterile, molecular grade water, and 1.5 μL of each primer (10 μM). Thermocycling conditions for PCR1 included a 98°C incubation step for 5 min, followed by 35 cycles of 98°C for 10 s, 58°C for 30 s, and 72°C for 30 s. Triplicate reactions for each sample were pooled and amplification was verified via gel electrophoresis. PCR1 products (55 μL) were size‐selected and cleaned with Ampure XP beads (Beckman Coulter) using a 0.75× bead: sample ratio, followed by a 1× bead: sample ratio, and confirmed via gel electrophoresis. Any samples containing non‐target bands post‐bead cleanup were subjected to size‐selection via gel extraction using the QIAquick gel extraction kit (Qiagen).

**TABLE 1:** Forward and reverse PCR1 primers and PCR2 indexes, including name, length, and original references, for the COX1 invertebrate DNA metabarcoding assay used in this study.

Name	Sequence (5′–3′)	Reference
LCO1490	**TCGTCGGCAGCGTCAGATGTGTATAAGAGACAG**GGTCAACAAATCATAAAGATATTGG	Jusino et al. (2019)
CO1‐CFMRa	**GTCTCGTGGGCTCGGAGATGTGTATAAGAGACAG**GGWACTAATCAATTTCCAAATCC	Jusino et al. (2019)
Index 1	CAAGCAGAAGACGGCATACGAGAT[10bp_Index]GTCTCGTGGGCTCGG	IDT‐Illumina UD Indexes
Index 2	AATGATACGGCGACCACCGAGATCTACAC[10bp_Index]TCGTCGGCAGCGTC	IDT‐Illumina UD Indexes

*Note*: PCR1 Illumina adaptor sequences are highlighted in bold.

PCR2 was performed in duplicate for each sample to add 10 bp barcodes to the 5′ and 3′ ends of each PCR1 product using IDT for Illumina UD Indexes (Table 1). PCR2 reactions used 25 μL volumes consisting of 2 μL of PCR1 template, 12.5 μL of KAPA HiFi HotStart ReadyMix, 5.5 μL of sterile, molecular grade water, and 2.5 μL of each IDT‐Illumina UD index primer. Thermocycling conditions for PCR2 included a 95°C incubation step for 3 min, followed by 10 cycles of 98°C for 30 s, 55°C for 30 s, and 72°C for 30 s. Duplicate reactions for each sample were pooled and amplification was verified via gel electrophoresis. PCR2 products (35 μL) were size‐selected and cleaned with Ampure XP beads (Beckman Coulter) using a 1× bead: sample ratio and confirmed via gel electrophoresis. PCR2 samples were normalized and pooled into sub‐libraries (*n* = 3) based on DNA concentrations determined using the Qubit dsDNA HS kit (Invitrogen). Ampure XP bead cleanups (1× bead: sample ratio) were performed again on each sub‐library to concentrate DNA and ensure non‐target amplicon removal prior to sequencing. Sub‐libraries were then normalized, pooled, and submitted for sequencing at the W. M. Keck Center (University of Illinois, Urbana, IL) on an Illumina NovaSeq 6000 using the SP flow‐cell with 2 x 250 bp paired‐end reads, with an output of 750 million reads.

### 2.4 Bioinformatics

Demultiplexed sequences were trimmed to remove adaptors from both 5′ and 3′ ends of each forward and reverse read using cutadapt (Martin, 2011) in Qiime2 v 2022.2 (Bolyen et al., 2019). Amplicon sequencing variants (ASVs) were generated using the DADA2 denoise function (Callahan et al., 2016) in Qiime2. Taxonomic assignments for each ASV were applied with the Qiime2 feature classifier classify‐sklearn function (Pedregosa et al., 2011) using a custom naive Bayes classifier trained on 1,005,220 unique invertebrate (and 43,356 vertebrate) COI sequences available on NCBI GenBank and BOLD (boldsystems.org). The invertebrate COI reference sequence database was curated to include all taxa within families known to occur in Wisconsin and neighboring states and trimmed to remove primers using the mkCOInr sequence database and pipeline developed by Megléz (Meglécz, 2023). To control for contamination, the maximum number of reads for each ASV found in field, extraction, and PCR controls were removed from all the other samples. All taxonomic classifications were confirmed via BLASTn search against the full GenBank nucleotide and BOLD databases. ASVs were considered misclassified if BLAST returned different identifications to species that occur in Wisconsin with greater than or equal to 98% pairwise identity. If more than one species was matched following this criterion, then the ASV was left identified to genus or family. For each sample, ASVs with three or more reads were retained and then combined by taxonomic classification to generate a list of taxa detected within each sample.

### 2.5 Statistical analysis

All statistical analyses were performed in R version 4.2.2 (R Core Team, 2022) and figures were produced using the *ggplot2* package (Wickham, 2016). We used detection/non‐detection data for gill lice eDNA from replicate water samples to simultaneously estimate occupancy (psi: the probability that eDNA of the target species is present at site *i*) and detection probability (*p*: the probability that eDNA of the target species is detected at time *i* at site *j*, given it was present at site *j*; MacKenzie et al., 2002). We used the *spOccupancy* package (Doser et al., 2022) to fit spatially explicit occupancy models in a Bayesian framework using Pólya‐Gamma data augmentation and Nearest Neighbor Gaussian Processes to account for spatial dependence (autocorrelation) among observations in detection/non‐detection data (Johnson et al., 2013). When constructing models, we set the hypermeans to 0 and the hypervariances to 2.72, which correspond to relatively flat priors on the probability scale and have been shown to be sufficient when specifying relatively uninformative (i.e., vague) priors (Doser et al., 2022). We specified two chains with 5000 samples per chain, a burn‐in of 3000, and a thinning rate of two for the Markov Chain Monte Carlo (MCMC) algorithm. We ensured adequate mixing of the MCMC chains occurred by inspecting Rhat values (which were <1.1 for all occupancy and detection estimates) and visually assessing trace plots (which showed constant and stable chain mixing). We ran intercept‐only models (i.e., models with no covariates) to estimate naïve occupancy and detection probabilities with 89% credible intervals (McElreath, 2018) of gill lice eDNA for each molecular assay (qPCR and metabarcoding). We conducted posterior predictive checks of both models by computing Bayesian *p*‐values using the Freeman‐Tukey statistic in *spOccupancy*. Bayesian *p*‐values less than .1 or greater than .9 may suggest lack of fit (Doser et al., 2022), but our models appeared to fit well (*p* = .33 for the qPCR data model and *p* = .34 for the metabarcoding data model).

We explored if read values from metabarcoding were predictive of *C*_*T*_ values from qPCR by fitting a linear mixed effects model using the *lme4* package (Bates et al., 2015). We treated reads as a fixed effect and sampling site as a random intercept to control for the non‐independence of multiple samples collected from the same site. For this analysis, we included only qPCR samples that were amplified (i.e., had a *C*_*T*_ value, *n* = 30). For plotting purposes, we generated predicted data for *C*_*T*_ values over the range of observed reads (0–986) using the *emmeans* package (Lenth, 2020). Because many samples had no reads for gill lice eDNA, we fit a zero‐inflated Poisson mixed effects model using the *GLMMadaptive* package (Rizopoulos, 2023) to determine if read counts were predictive of the number of qPCR technical replicates that amplified per sample (“qPCR score” of 0–3). We treated qPCR score as a fixed effect and site sampling site as a random intercept (as above).

## 3 RESULTS

### 3.1 eDNA metabarcoding versus qPCR

**FIGURE 3:**
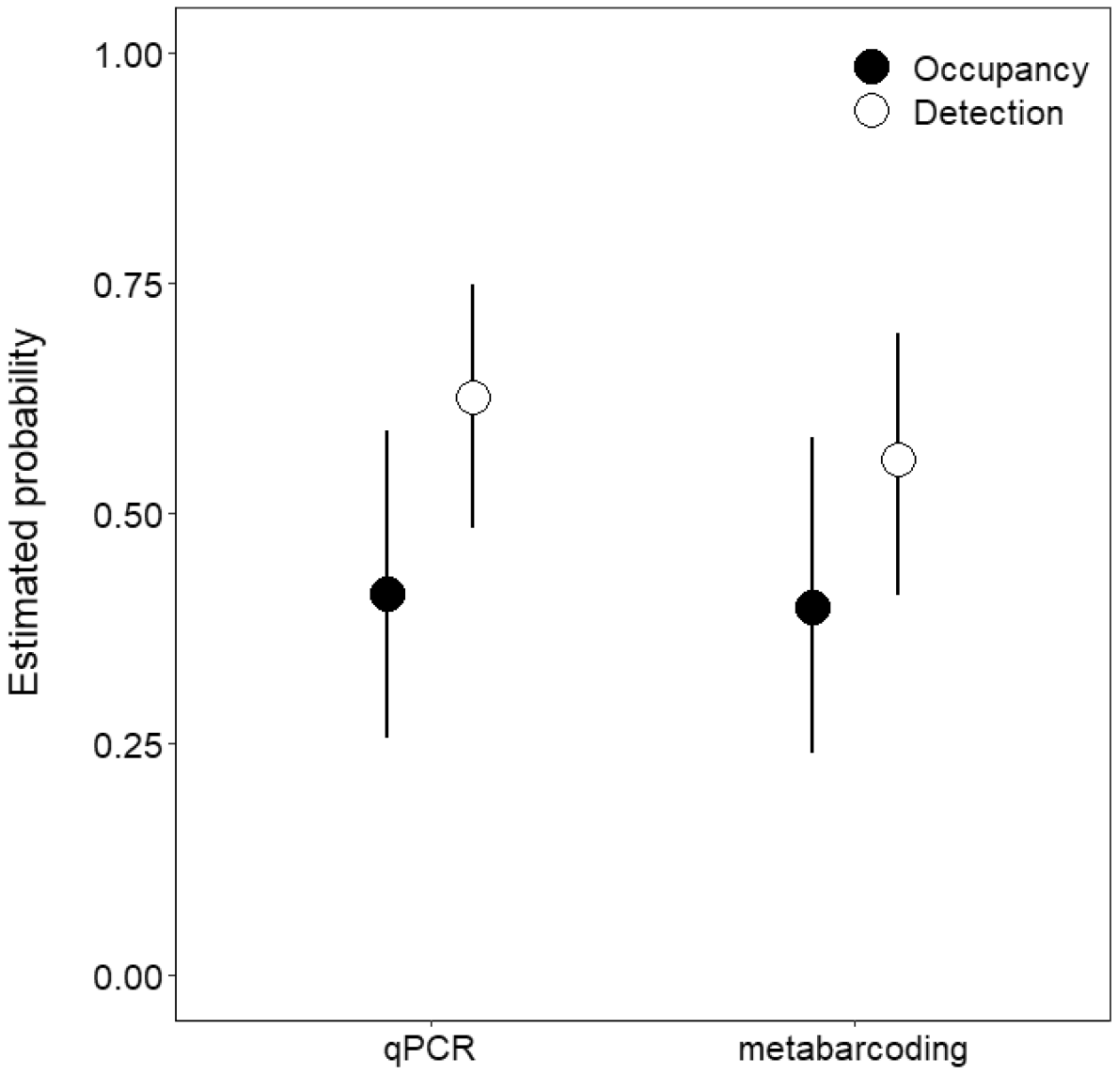
Occupancy and detection probability estimates with 89% Bayesian credible intervals for gill lice (*Salmincola edwardsii*) environmental DNA from streams on Fort McCoy, Wisconsin, USA based on molecular assay (qPCR or metabarcoding).

**FIGURE 4:**
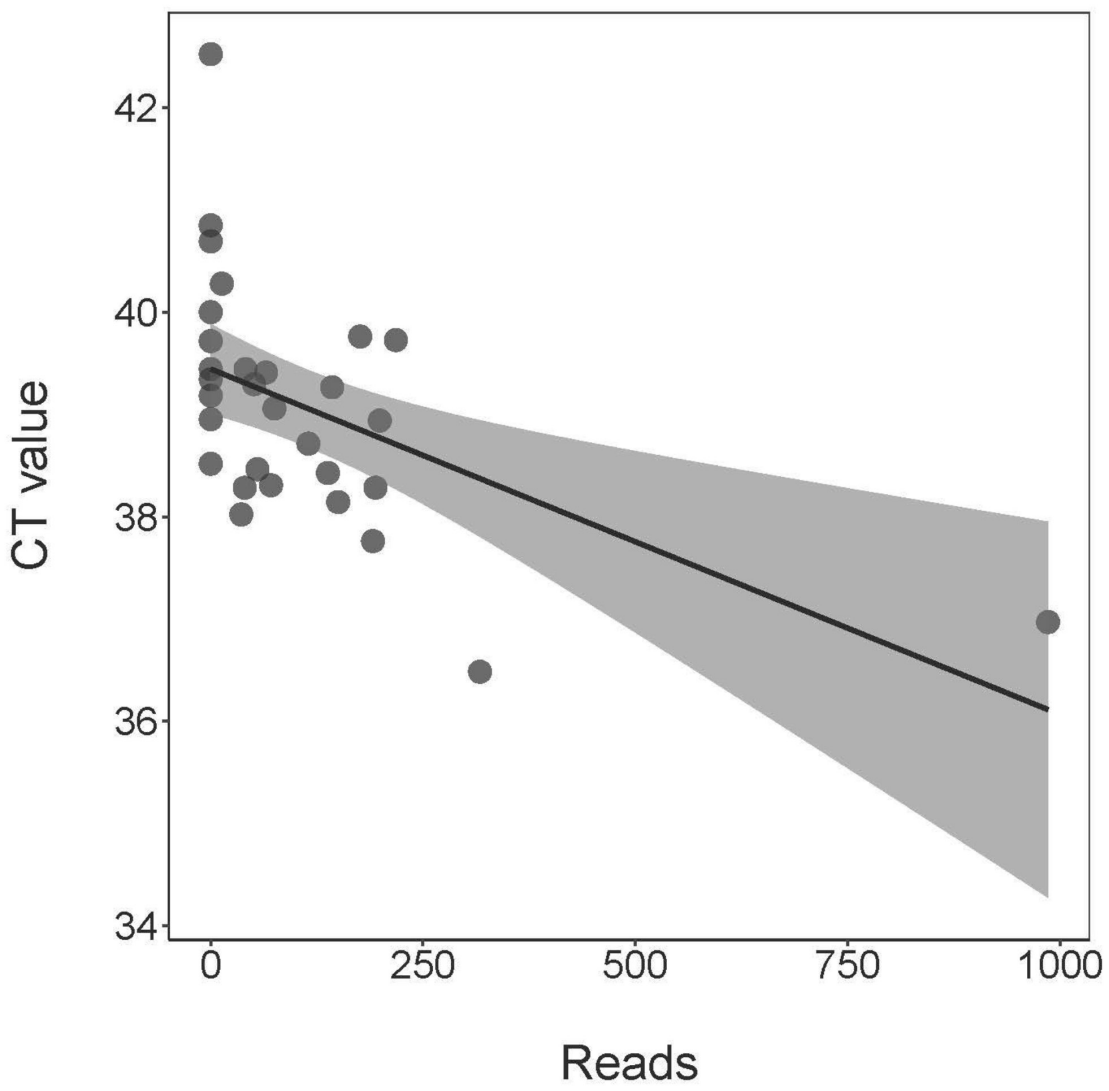
Predicted *C*_*T*_ values from qPCR as a function of reads from metabarcoding for detection of gill lice (*Salmincola edwardsii)* environmental DNA from streams on Fort McCoy, Wisconsin, USA. Note that as *C*_*T*_ values decrease, the number of reads increases.

We compared qPCR (Katz et al., 2023) and metabarcoding for the detection of *S. edwardsii* across 114 samples collected from 38 stream sites. Metabarcoding analysis detected *S. edwardsii* at 14 sites (26 samples), while Katz et al. (2023) detected *S. edwardsii* at 15 sites (30 samples) with qPCR (Figure 2; Data S1). eDNA concentrations were reported to be below the limit of quantification (Katz et al., 2023), indicating eDNA concentrations would have unacceptable variability and a lack of precision, leading us to utilize *C*_*T*_ values instead (Katz et al., 2021; Klymus et al., 2020). We found no evidence suggesting gill lice occupancy or detection probabilities significantly differed between qPCR and metabarcoding, as point estimates and credible intervals for both molecular assays were very similar for these parameters (Figure 3). In total, our metabarcoding analysis detected 3710 reads for *S. edwardsii*. *Salmincola edwardsii r*eads from metabarcoding were reasonably predictive of and negatively associated with qPCR *C*_*T*_ values (*β* = −.0023, 95% CI: −0.0045 to −0.0003; *p* = .03), although confidence intervals were wide at the largest predicted values of *C*_*T*_ because we lacked many observed read values >300 for generating predictions (Figure 4). We found no evidence to suggest reads were predictive of qPCR scores, as the 95% confidence interval for the parameter estimate for reads included zero (0.0009, 95% CI: −0.0003 to 0.0021; *p* = .15).

### 3.2 eDNA metabarcoding diversity

We detected a total of 2691 taxa totaling 50,863,905 reads across all 38 sites. These 2691 taxa included 1847 species, 1597 genera, 472 families, 23 classes, 59 orders, and 10 phylum. Among these taxa, Arthropoda had the highest reads and ASVs, followed by Porifera and Annelida (Data S1). Arthropoda had the vast majority of both reads (72%) and ASVs (94%). We also investigated the frequency of detection for specific taxa with two metrics: read number and frequency (proportion of sites a given taxon was detected at). *Simulium* (black flies) followed by Spongillida (freshwater sponges) and Chironomidae (Non‐biting midges) had the three highest percentages of reads. There were eight ASVs that were found across every sample, two families, Cecidocmyiidae (gall and wood midges) and Chironomidae (non‐biting midges), five genera, *Simlium* (black flies), *Corynoneura* (non‐biting midges), *Peltasta* (twirler moth), *Lecane* (monogonont rotifers), and *Adineta* (bdelloid rotifers), and one species, *Rotaria rotatoria* (pale‐white bdelloid rotifer). Contamination from our field, extraction, and qPCR blanks was minimal with only 560 reads (.001%) across 29 ASVs (1.0%), resulting in the removal of only three taxa after normalization (*Typhaea stercorea*; *Idionella_rugosa*; *Salvelinus_fontinalis*).

While an in‐depth analysis of all the species that were detected within our metabarcoding analysis is outside the scope of this study, we did detect a wide diversity of both freshwater parasites and terrestrial parasitoids. For example, one of the most common types of parasites we detected were leeches, including the species *Dina parva* and *Haemopis marmorata*. Broadly, leech eDNA (Hirdinea) was detected at 79% of our sites. We also detected numerous other organisms such as the parasitoid wasp *Ceracia dentata*, multiple ASVs within the parasitoid wasp families of Ichneumonidae and Braconidae, and parasitic nematodes (*Steinernema glaseri*; Data S1).

## 4 DISCUSSION

To determine whether eDNA metabarcoding was a viable tool for aquatic parasite surveillance, we compared qPCR with metabarcoding for the eDNA detection of the ecologically and economically damaging gill louse *S. edwardsii*. We found that there was no evidence to suggest occupancy or detection probability of *S. edwardsii* differed between qPCR and metabarcoding, suggesting that inferences of gill lice occupancy at Fort McCoy are unaffected by molecular methodology. While there were no statistical differences in detection probabilities, we did observe small differences in site and sample level detections between qPCR and metabarcoding: two sites had *S. edwardsii* eDNA detections only via qPCR and one site only detected *S. edwardsii* eDNA with metabarcoding. Further, qPCR analysis in Katz et al. (2023) detected *S. edwardsii* eDNA in 30 samples compared to 26 detected in this study via metabarcoding. These discrepancies could be the result of several factors. For example, differences in methods (e.g., single vs. multiple rounds of PCR, enzyme‐ vs. bead‐based cleanups), PCR reagent types and concentrations (e.g., of enzymes, primers), primer specificity and binding efficiency (e.g., for single vs. multispecies targets), PCR reaction conditions (e.g., annealing temperatures, cycle times), and starting DNA template volumes (e.g., 3 vs. 5 μL for qPCR and metabarcoding, respectively) could be associated with the observed differences in site and sample level eDNA detections between qPCR and metabarcoding. It is also possible that the differences could be due to stochastic variability that is inherent with all sampling methods, especially when targeting species with low‐densities/abundances, or in this case, when targeting low‐concentration eDNA. Moving forward, it is vital to explore how to minimize such variability. For instance, increased field and technical replicates can help to limit methodological biases and stochastic error associated with eDNA analysis (Buxton et al., 2021).

We also found metabarcoding reads to be negatively predictive of *C*_*T*_ values from qPCR for *S. edwardsii* (Figure 4). This is reasonable because as the number of *S. edwardsii* reads increase within a sample the *C*_*T*_ value should decrease, indicating higher *S. edwardsii* eDNA starting concentrations. This relationship can also be helpful to determine if there is any primer bias occurring throughout the amplification process (Kelly et al., 2019). For example, if the metabarcoding primers were not effectively amplifying (or biased against) *S. edwardsii* eDNA, the number of reads would either show no relationship with *C*_*T*_ values or be relatively low for samples with low *C*_*T*_ values. However, we also found that the number of reads was not predictive of overall qPCR score for *S. edwardsii*, indicating that an increase in DNA per sample did not relate to consistent detections across qPCR replicates. This result was shared by Biggs et al. (2015) who found that qPCR score was not indicative of great crested newt (*T. cristatus*) abundance. Conversely, Harper et al. (2018) found their metabarcoding results were indicative of qPCR score and eDNA concentration for great crested newts, though this study used four times the number of qPCR replicates, possibly allowing for increased statistical power. Overall, the differences in the literature, combined with our results showing reads were predictive of *C*_*T*_ value but not qPCR score, highlight the variability that requires future study to determine how species ecology and eDNA ecology (Barnes & Turner, 2016) may influence eDNA detection and read counts. Future studies should expand upon our work to explore the relationship between *S. edwardsii* metabarcoding reads and species abundance, gill louse phenology, life stage, and ecology. This would be impactful for *S. edwardsii* where levels and impacts of infection for native brook trout populations are primarily determined by the concentration of *S. edwardsii* in the system (Mitro, 2016). The conclusions from this study should be assessed across environments and target species, as oftentimes such environmental and species changeability leads to varied results (Biggs et al., 2015; Harper et al., 2018).

In addition to finding that metabarcoding and qPCR performed similarly in predicting *S. edwardsii* occupancy and detection, our metabarcoding analysis inherently produced a large amount of invertebrate and parasitic community detection data that would be impossible to replicate with qPCR. Our metabarcoding analysis identified 2691 taxa (1847 identified to species) including multiple terrestrial and aquatic parasites and parasitoids. This is particularly meaningful because the detection and identification of aquatic parasites is typically both challenging and time‐consuming (Li et al., 2022). Oftentimes, choosing between metabarcoding and qPCR requires a tradeoff that needs to be made for increased taxonomic coverage or sensitivity. With an optimized assay, eDNA metabarcoding technology could be used to create an inventory of all parasites present in a system, allowing for a more holistic and proactive management approach while broader invertebrate data could be leveraged to explore the influence of community ecology on parasite or host species detection or track how changing communities impact eDNA signals over time (Bakker et al., 2017; Johnson, Fokar, et al. 2021; Xie et al., 2018). Such multi‐use approaches would allow for in‐depth analyses on community dynamics while pursuing classic presence/abundance studies.

Overall, we found that there is no evidence to suggest that detections differed between qPCR and metabarcoding approaches. We also found that our eDNA metabarcoding *S. edwardsii* reads significantly predicted qPCR *C*_*T*_ values though were not predictive of qPCR score, underscoring the need for additional research on how *S. edwardsii* ecology, abundance, and life history influences their eDNA detection. We also demonstrated how eDNA metabarcoding can be used to effectively detect a freshwater parasite of conservation interest while and at the same time, identify thousands of species that comprise the broader invertebrate community. We suggest that future studies should utilize these high‐resolution and comprehensive community‐level eDNA metabarcoding data to explore how gill lice are impacted by changes in their broader invertebrate community. Lastly, we highlight the need for conducting similar analyses across environments and target species, as the ecology of eDNA will vary on a per‐study basis. In combination, our results suggest that eDNA metabarcoding is a viable alternative to qPCR for the detection of gill lice parasites, but unlike qPCR, it can offer additional management utility by providing novel opportunities to address broader community‐level ecological questions.

## AUTHOR CONTRIBUTIONS

**Mark Johnson:** Data curation (equal); formal analysis (equal); writing – original draft (equal); writing – review and editing (equal). **Sasha Tetzlaff:** Conceptualization (equal); data curation (equal); formal analysis (equal); methodology (equal); writing – review and editing (equal). **Aron Katz:** Conceptualization (equal); data curation (equal); formal analysis (equal); investigation (equal); methodology (equal); validation (equal); writing – review and editing (equal). **Jinelle Sperry:** Conceptualization (equal); funding acquisition (equal); methodology (equal); project administration (equal); writing – review and editing (equal).

## CONFLICT OF INTEREST STATEMENT

The authors declare no competing interests.

## Supporting information


Data S1.

## Data Availability

The raw sequencing data used within this experiment will be archived with the Dryad data repository once accepted.
